# Changes in Food Purchasing Practices of French Households During the First COVID-19 Lockdown and Associated Individual and Environmental Factors

**DOI:** 10.3389/fnut.2022.828550

**Published:** 2022-03-03

**Authors:** Daisy Recchia, Pascaline Rollet, Marlène Perignon, Nicolas Bricas, Simon Vonthron, Coline Perrin, Caroline Méjean

**Affiliations:** ^1^MoISA, Univ Montpellier, CIRAD, CIHEAM-IAMM, INRAE, Institut Agro, IRD, Montpellier, France; ^2^CIRAD, UMR MoISA, Montpellier, France; ^3^INNOVATION, Univ Montpellier, CIRAD, INRAE, Institut Agro, Montpellier, France

**Keywords:** COVID-19 lockdown, food purchasing behaviors, grocery shopping, food outlets, food environment, France

## Abstract

**Background:**

To limit the spread of COVID-19, a strict lockdown was imposed in France between March and May 2020. Mobility limitations and closure of non-essential public places (restaurants, open-air markets, etc.) affected peoples' food environment (FE) and thus their food purchasing practices (FPPs). This study aimed to explore changes in FPPs of French households during lockdown and associations with individual and environmental factors.

**Methods:**

In April of 2020 households from the Mont'Panier cross-sectional study (*n* = 306), a quota sampling survey conducted in the south of France, were asked to complete an online questionnaire about their FPPs during lockdown and related factors, including perceived FE (distance to closest general food store, perception of increased food prices, etc.). Objective FE (presence, number, proximity, and density of food outlets) was assessed around participant's home using a geographical information system. Multiple correspondence analysis based on changes in frequency of use and quantity of food purchased by food outlet, followed by a hierarchical cluster analysis, resulted in the identification of clusters. Logistic regression models were performed to assess associations between identified clusters and household's sociodemographic characteristics, perceived, and objective FE.

**Results:**

Five clusters were identified. Cluster “Supermarket” (38% of the total sample), in which households reduced frequency of trips, but increased quantity bought in supermarkets during lockdown, was associated with lower incomes and the perception of increased food prices. Cluster “E-supermarket” (12%), in which households increased online food shopping with pickup at supermarket, was associated with higher incomes. Cluster “Diversified” (22%), made up of households who reduced frequency of trips to diverse food outlet types, was associated with the perception of increased food prices. Cluster “Organic Food Store” (20%), in which households did not change frequency of trips, nor quantity purchased in organic food stores, was associated with being older (35–50 y vs. <35 y). Finally, cluster “Producer” (8%), which includes households who regularly purchased food from producers, but mostly reduced these purchases during lockdown, was associated with the presence of an organic food store within a 1-km walking distance around home.

**Conclusion:**

This study highlighted diverse changes in FPPs during lockdown and overall more significant associations with perceived than with objective FE indicators.

## Introduction

The coronavirus disease 2019 (COVID-19) pandemic induced lockdowns in several parts of the world, which include France, where governmental authorities imposed a strict lockdown between March 17 and May 10 to slow down and contain the spread of the virus. During this lockdown, the French population was not allowed to leave home except for essential activities such as grocery shopping, medical appointments, legal obligations, and physical activity practices of short duration in the vicinity of home. All non-essential public places and businesses were temporarily closed, which constrained a large part of the working population to work from home or to be temporarily unemployed (partial/technical unemployment). The pandemic's economic impact was thus two-sided, with on one side income drops and on the other side increased savings due to decreased spending on cultural or leisure activities. These shifts in purchasing power, along with other consequences of the pandemic, such as mobility limitations and closure of restaurants, takeaways, canteens, and open-air markets suddenly disrupted people's daily routines, which includes their food shopping habits.

This unprecedented situation stimulated the interest of researchers in the influence of COVID-19 lockdowns on food habits. Changes toward healthier and changes toward less healthy eating behaviors were found in studies conducted worldwide (1). In Europe changes included increased fruit and/or vegetable (FV) consumption (2, 3) and more home-cooked meals (3, 4). Meanwhile, snacking (2, 5) and increased consumption of comfort foods (e.g., energy dense, ultra-processed, sweet and savory foods, alcohol) (5–7) were found to be part of the consequences of the lockdown. Both unhealthy and healthy changes in dietary habits during the lockdown were also found in multiple studies conducted in France (8–12).

Changes in food purchasing behaviors were also studied, but to a lesser extent. Panic buying and stockpiling of food products were observed in many parts of the world (13). Reduced grocery shopping frequency, increased online shopping, and increased purchasing of foods with longer shelf life, such as dried, canned, and frozen foods, were the most common observed changes (14–16). Less is known about consumer's choice of food outlet type during lockdown. The International Agricultural Trade Research Consortium (IATRC) stated that supermarket sales went up at the costs of other retail outlets as shopping trips were less frequent and individual consumers concentrated most purchases on one shop (17). In France, most frequented food outlets during the lockdown were supermarkets and bakeries, with however decreased frequency of use during the lockdown; meanwhile, internet-, phone-, or mail-ordered purchases increased, and frequency of use of local open-air markets reduced drastically due to their closure during the lockdown in cities with more than 20, 000 inhabitants (8). Meanwhile, short food supply chains appear to have been reassuring citizens during the COVID-19 crisis by promoting food sovereignty and increasing food security (18, 19).

The lockdown most likely influenced people unevenly across France according to location (population density, number of COVID-19 cases), but also according to the socioeconomic level of regions. Pullano et al. assessed the effect of demographic and socioeconomic factors during the lockdown in France and reported stronger mobility drops in highly affected regions, but moderate associations with the socioeconomic level of regions (20).

Given the closure of away-from-home food services (restaurants, fast-foods, takeaways, canteens, etc.) and open-air markets, COVID-19 lockdown further changed people's built food environment (FE), which can be defined as the physical distribution of food sources (objective FE). The perceived FE, which is alternatively characterized as consumer's experience of the FE, including in-store experience, was likewise altered during the lockdown due to the variability of food prices (21) and consumer's perceived changes in produce availability (22, 23). Mobility restrictions constraining people to stay in a given perimeter around their home probably nudged consumers to use food outlets in their living area. Consequently, changes in food shopping practices during lockdown might be associated with the FE around people's home. To our knowledge, no prior study has investigated the associations between changes in food purchasing practices (FPPs) and/or objective FE.

Accordingly, we aimed to explore changes in FPPs of southern French households during the first COVID-19 lockdown and assess the associations between these changes and related individual and environmental factors. To do so, we identified clusters based on changes in the organization of grocery shopping practices, namely frequency of use and quantity of food purchased by food supply source. Then, we investigated the associations between clusters and households' socioeconomic and demographic characteristics and also environmental factors, such as the perceived and the objective FE.

## Methods

### Study Population

In April of 2020, participants of the Mont'Panier cross-sectional study were asked to complete an online questionnaire about their FPPs and other related factors during the first COVID-19 lockdown. Briefly, the Mont'Panier study (https://www.etude-montpanier.com) was carried out from May 2018 to December 2019 among households living in the south of France. To be included in the Mont'Panier study, participants had to be 18 or older and live in the Montpellier Metropolitan Area (MMA). Using sociodemographic data of the MMA from the French National Institute of Statistics (INSEE), quota sampling was performed based on household composition (one adult, multiple adults, one adult with at least one child, and multiple adults with at least one child) crossed with age of household head (<30, 30–50, and > 50 years). This study was conducted according to the guidelines laid down in the Declaration of Helsinki, and all procedures were approved by the Institutional Review Board of the French Institute for Health and Medical Research (IRB Inserm n° IRB00003888 IORG0003254 FWA00005831) and were registered to the Commission Nationale Informatique et Libertés. Written electronic informed consent to participate in the study was obtained after a thorough explanation of the study to each of the participants. Participants received a 15 € voucher for returning all data collection materials duly completed.

### Lockdown Questionnaire

Participants filled in the COVID-19 lockdown specific online questionnaire, which was launched in April 2020 and included multiple questions on changes in FPPs and related factors. Changes in frequency of use of food supply sources and quantity of food purchased by food outlet were assessed, as were the reasons for these changes. Food supply sources that were considered included supermarkets, e-supermarkets (online food purchasing with pickup at supermarket, called *drives* in French), markets (open-air and closed), organic food stores, greengrocers, other specialized food stores (bakeries, butcher's, fishmonger's, dairy stores etc.), small grocery stores, discount food stores, frozen food stores, and direct sales from producers (basket orders with home delivery or pickup at the farm or drop-off-location). Producers include FV growers (called *maraîchers* in French), farmers and Associations for the Maintenance of Peasant Agriculture (AMAP), which is a French version of Community Supported Agriculture (CSA).

### Covariates

Socioeconomic and demographic characteristics were obtained through the online questionnaire of the Mont'Panier study (May 2018 to December 2019) and through the COVID-19 lockdown specific questionnaire (April 2020). Income per unit of consumption (quartiles of the MMA: <980, 980–1,722, 1,723–2,550, and >2,550 €/month), household head's age group (<35, 35–50, >50 years), and level of education (high school degree or lower, undergraduate degree, and postgraduate degree) were obtained through the online questionnaire of the Mont'Panier study. Meanwhile, household composition (one adult, multiple adults, one adult with at least one child, and multiple adults with at least one child) and reported drop of income during lockdown were obtained from the COVID-19 lockdown-specific questionnaire. Median income, by IRIS (“Aggregated unit for Statistical Information”) for households living in the cities of Montpellier, Lattes, Juvignac, Castelnau-le-Lez, and Mauguio or by municipality for households living in other cities of the MMA, was used to take into account neighborhood income level. French IRIS areas are the preferred fundamental administrative unit, used by the French national institute for statistics and economic studies (INSEE) for the dissemination of infracommunal data.

Household's food purchases, which were assessed in the Mont'Panier study over a 1-month period using food supply diaries and grocery receipts, allowed us to calculate share of expenses by food supply source before lockdown.

### Perceived and Objective Food Environment

The perceived FE was assessed using questions from the lockdown specific online questionnaire, namely perceived walking distance to the closest general food store from home and perceived variability of food prices. Reasons for changes in FPPs declared in the questionnaire were also used as perceived FE variables, namely, buying local products, in-store availability of food products, and store accessibility (closure, public transportation, and parking facilities, etc.).

The objective FE was assessed around participants' home, using the localization of food outlets in the study area and participants' home addresses. Participants' home addresses were obtained through the online questionnaire of the Mont'Panier study, and the localization of food stores in the study area was obtained through the national identification system for natural and legal persons and their establishments (SIRENE) database from INSEE. The SIRENE database was completed, corrected, and verified using: (i) OpenStreetMaps, which provides open data of companies and establishments, through a collaborative project in which external contributors can update and enrich the database, (ii) online searches on Google Maps, company websites of major food retailers, and city websites (e.g., with information on local markets), and (iii) field observations of about 10% of the studied area.

Classification of food outlet types was done based on the initial classification of food stores of the SIRENE database. In this study, we focused on multiple types of food outlets: supermarkets, markets (open-air markets and covered markets), greengrocers, bakeries, other specialized food stores (butcher's, fishmonger's, and dairy stores), organic food stores, and small grocery stores.

Geographical information systems were performed to calculate FE indicators around participants' home using QGIS (version 3.4.7.). Four types of indicators estimated the objective FE around participants' home: number, presence, proximity, and relative density. The proximity of food stores was calculated by assessing the shortest road network distance between the nearest food outlet relative to each home address. The number of each food outlet type was calculated within a 1,000-m road network buffer around each home address. A 1,000-m buffer was chosen since the French population had to stay within a 1 km radius of their home during the lockdown except for essential activities. The number of food outlets was used to calculate the presence (binary count) and the relative density of food stores (e.g., relative density of food stores selling FV = number of food outlets selling FV/the total number of food outlets). A number of food outlet variables were categorized into three groups for main analysis given their non-normal distribution.

### Statistical Analysis

Descriptive statistics were expressed as percentages and means (standard deviation). Differences between clusters were assessed by Pearson's chi-square tests for categorical variable and Wilcoxon tests for numerical variables.

To identify different patterns of change of FPPs during lockdown, we used a two-step procedure. First, a multiple correspondence analysis (MCA) was applied on changes in frequency of use of food supply sources and changes in quantity of food purchased by food outlet type. Inertia, that is, the variance in individual patterns around the average pattern, is measured. MCA decomposes the inertia by identifying a small number of mutually independent dimensions (24). Dimensions are formed by identifying the axes for which the distance between the patterns and axes is minimized, while simultaneously maximizing the amount of explained inertia. Each dimension has an eigenvalue, and the ratio of the eigenvalue for one dimension represents the proportion of the total inertia explained by that dimension. The number of retained dimensions is chosen using Kaiser Criterion, to obtain a cumulative percentage of acceptable variance (25). Using the dimensions retained, a clustering procedure was then performed by applying Ward's hierarchical classification of the individuals, followed by K-means clustering, maximizing the interclass inertia. The graphical observation of the dendrogram, which illustrates stages of classification, was used to estimate the appropriate number of clusters (26). Stabilization of the clusters was carried out to distribute the individuals better by clusters. Cluster analysis yielded groups, interpreted as patterns of changes in the organization of FPPs, labeled according to their frequency of use and quantity of food purchased by food outlet.

Logistic regression models were performed by calculating odds ratios (ORs) and 95% confidence intervals (CIs) to determine the strength of the associations between each cluster membership (belonging to this cluster vs. not) and each explanatory variable, that is, socioeconomic and demographic characteristics, and also perceived and objective FE variables. The equation of the logistic regression models was as follows: Y = β_0_ + β_1_X_1_ + … + β_p_X_p_, where Y = binary variable for belonging to cluster X (0/1), β_0_ = intercept, X_1−p_ = individual and environmental variables and β_1−*p*_ = coefficients for the corresponding variables. Only explanatory variables associated with clusters at 0.1 significance level in bivariate analyses were retained for inclusion in the subsequent multivariate models. Subsequently, multivariable backwards-stepwise logistic regression was performed to determine the variables included in the final model, with income per unit of consumption, household composition, age, and educational level of household head forced into the model. Variables whose exclusion from the model caused large fluctuations in OR (>10%), and also variables whose exclusion increased the significance of the likelihood ratio tests (*p* > 0.05), were re-entered into the model.

Analyses were conducted on a weighted sample where weights were calculated by raking ratio so that the marginal distribution of the weighted sample conforms to the marginal distribution of the targeted population. Data on socioeconomic and demographic characteristics of the MMA were obtained from the INSEE database of 2017. The sample was adjusted by calibration on margins based on income per unit of consumption and household composition crossed with household head's age group. All analyses presented in this paper were conducted on the weighted sample.

Statistical analyses were performed using R Statistical Software (version 4.1.0), and the threshold for statistical significance was *p* < 0.05.

## Results

Analyses were carried out on 306 households residing in the MMA, who had previously participated in the Mont'Panier study (May 2018 to December 2019), responded to the lockdown specific online questionnaire (April 2020) and did not change place of residence during the lockdown.

Given the sample's adjustment by calibration on margins based on socioeconomic and demographic characteristics of the MMA population, distributions correspond to those of the real population. These results are presented in the first column of Table 1. Two-thirds of households were households without children, composed of a single adult or multiple adults, quartiles of income per unit of consumption were 980, 980–1,722, 1,723–2,550, and 2,551 €/month, nearly half of household heads were over 50 years old, and most household head had an educational level higher than high school degree.

**Table 1:** Households' socioeconomic and demographic characteristics.

	**Total sample^a^**	**Cluster** **Supermarket 38%**	**Cluster** **E-supermarket^b^ 12%**	**Cluster** **Producer^c^ 8%**	**Cluster Organic** **Food Store 20%**	**Cluster** **Diversified 22%**	**Pearson's** ***X*^2^ *p*-value**
**Household composition**							**0.011**
One adult	**43.8%**	**52.2%**	36.3%	21.9%	**41.3%**	**43.4%**	
Multiple adults	22.8%	22.9%	15.6%	22.4%	29.4%	20.9%	
One adult with at least one child	11.9%	13.7%	0.0%	**34.2%**	10.0%	9.3%	
Multiple adults with at least one child	21.5%	11.3%	**48.1%**	21.6%	19.4%	26.4%	
**Income per consumption unit**							**0.016**
<980 €/month	25.0%	**38.1%**	9.5%	**31.1%**	11.1%	21.4%	
980–1,722 €/month	25.0%	25.9%	**42.9%**	28.9%	19.2%	17.1%	
1,723–2,550 €/month	25.0%	21.7%	22.7%	15.8%	32.5%	28.6%	
≥2,551 €/month	25.0%	14.3%	25.0%	24.3%	**37.3%**	**32.9%**	
**Age of household head**							0.279
<35 years	28.7%	36.7%	25.5%	31.8%	13.7%	28.7%	
35–50 years	26.5%	18.7%	**41.2%**	23.2%	32.0%	28.0%	
>50 years	**44.9%**	**44.6%**	33.4%	**45.0%**	**54.3%**	**43.3%**	
**Level of education of household head**							0.055
High school degree or lower	13.8%	17.9%	13.4%	2.1%	10.9%	13.5%	
Undergraduate degree	42.7%	**49.9%**	**44.6%**	**60.2%**	38.7%	26.5%	
Postgraduate degree	**43.5%**	32.2%	42.0%	37.7%	**50.4%**	**60.1%**	

a *The sample was adjusted by calibration on margins based on income per unit of consumption and household composition crossed with household head's age group.*

b *E-supermarket: Online food shopping with pickup at supermarket (called drive in French).*

c *Producer: direct sales from producers [e.g., fruit and vegetable growers (called maraîchers in French), farmers, basket orders from Associations for the Maintenance of Peasant Agriculture (AMAP), which is a French version of Community Supported Agriculture].*

*The numbers in bold represent the highest percentages among each cluster for each variable*.

### Changes in Food Purchasing Practices During the Lockdown

**Figure 1:**
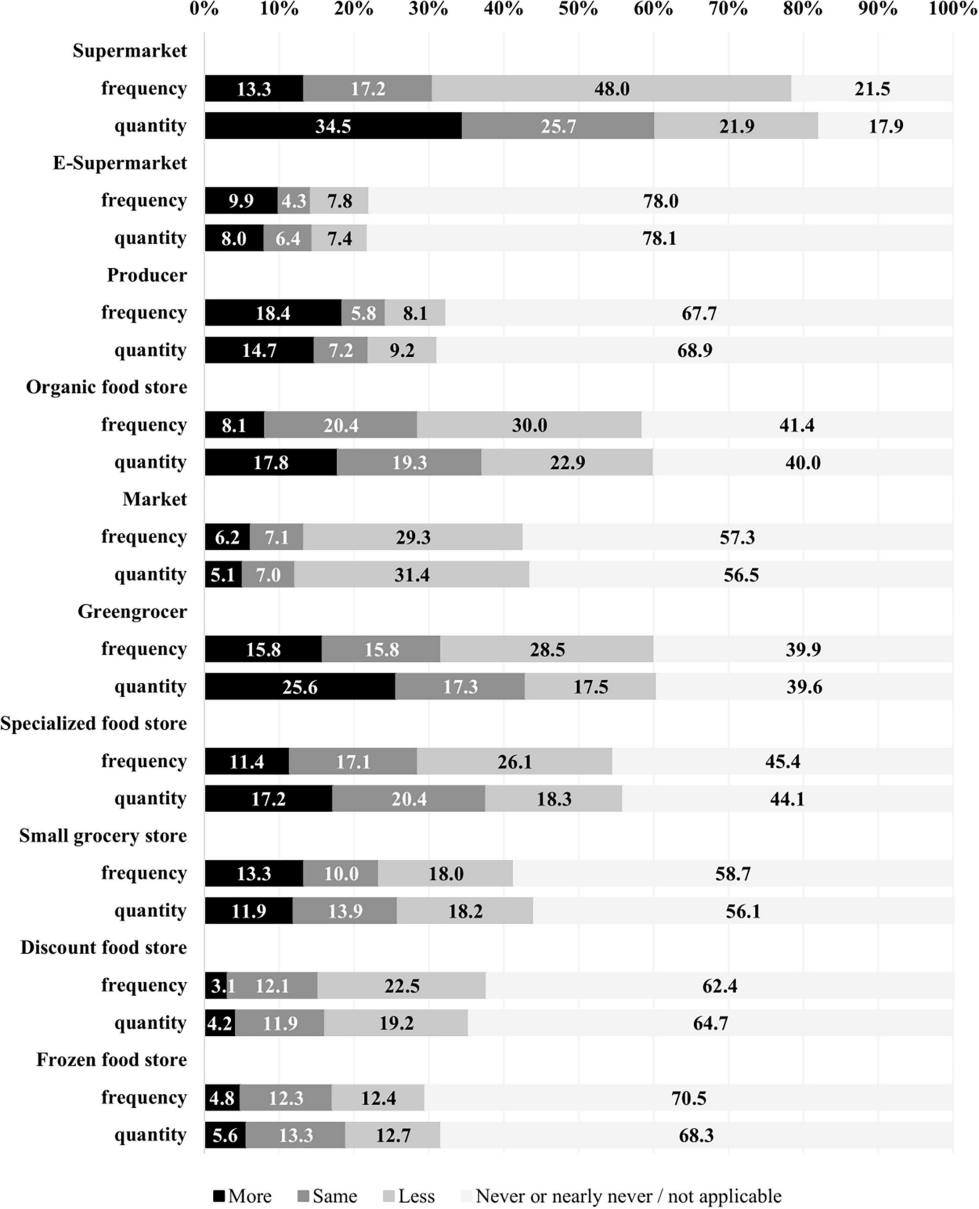
Changes in frequency of use and quantity purchased by food supply source during lockdown. E-supermarket: online food shopping with pickup at supermarket (called drive in French). Producer: direct sales from producers [e.g., fruit and vegetable growers (called maraîchers in French), farmers, basket orders from Associations for the Maintenance of Peasant Agriculture (AMAP), which is a French version of Community Supported Agriculture]. Specialized food store: bakeries, butcher's, fishmonger's, and dairy stores.

Changes in the frequency of use of food supply sources and quantity of food purchased by food supply source during lockdown are regrouped in Figure 1. Nearly half of the weighted sample reduced their frequency of use of supermarkets and one-third increased quantity of food purchased in supermarkets. Online grocery shopping with pickup at supermarket increased in frequency of use and also in quantity purchased for about one in 10 households. Increased frequency of purchases from producers was found for about two in 10 households. Mostly decreased frequency of visits and also decreased quantity of food purchased were observed for other food outlet types, with the exception of greengrocers, where an increase in quantity of food purchased occurred for about one-fourth of households.

It should be noted that percentages presented as “more” for frequency of use and also include households who reported using the respective food supply sources only since the lockdown. For instance, among the households that reported increased frequency of use of e-supermarkets, half (5%) were new users of e-supermarkets. Likewise, 6.5% of households reported being new users of supermarkets and hard-discount stores combined. We also observed 0.5% of new users of organic food stores and 8.5% for producers.

Supplementary Table 1 presents description of main reasons for change in FPPs during lockdown, perceived FE, and other related factors for the total weighted sample (column 1). Results by cluster will not be described here to avoid redundancy, but are available in columns 2–6 of Supplementary Table 1.

### Main Reasons for Change in Food Purchasing Practices During Lockdown

Main reasons for change in FPPs during lockdown were “limiting exposure to the COVID-19 virus” (66.4%) and to a lesser extent “changes in cooking and consumption” (34.1%), “distance to food store” (31.1%), “in-store availability of food products” (29.7%), “store accessibility” (29.4%), and “buying local products” (27.8%) (Supplementary Table 1).

### Perceived Food Environment

Walking distance from home to the closest general food store was under 15 min for 70% of the weighted study sample and 11% reported needing more than 30 min to get to the closest general food store. One-third of the weighted study sample perceived a rise in food prices during the lockdown, whereas nearly half of the sample reported not knowing whether prices of food products increased or not (Supplementary Table 1).

### Other Related Factors

Half of households of the weighted study sample reported increased grocery expenses, more than two-thirds of households reported no stockpiling of food products and 72% reported a drop of household income during lockdown (Supplementary Table 1).

### Clusters of Change of Food Purchasing Practices During Lockdown

Five clusters were identified and interpreted as changes in FPPs as “*Supermarket*,” “*E-supermarket*,” “*Producer*,” “*Organic food store*,” and “*Diversified*,” representing, respectively 38, 12, 8, 20, and 22% of households of the total sample.

Table 1 presents socioeconomic and demographic characteristics of households for each cluster (columns 2–6), and Table 2 presents mean share of expenses before lockdown by food supply source for each cluster. Changes in frequency of use and quantity of food purchased by food supply source for each cluster are represented in Supplementary Table 2.

**Table 2:** Mean share of expenses before lockdown by food supply source for each cluster.

	**Cluster Supermarket 38%**	**Cluster E-supermarket^a^ 12%**	**Cluster Producer^b^ 8%**	**Cluster Organic Food Store 20%**	**Cluster Diversified 22%**	**Wilcoxon/ Mann-Whitney *p*-value**
Supermarket	**56.02 (4.03)**	**57.28 (5.81)**	**46.38 (8.96)**	**43.87 (3.10)**	**46.06 (2.96)**	0.063
Discount food store	**19.08 (3.43)**	**14.15 (7.75)**	2.23 (1.75)	3.39 (0.80)	5.87 (1.34)	**<0.001**
Small grocery store	6.68 (2.41)	8.63 (3.25)	**10.00 (4.65)**	8.83 (2.05)	8.62 (2.03)	0.064
Producer^b^	1.12 (0.26)	1.42 (0.55)	**10.24 (4.91)**	3.63 (1.19)	3.16 (0.80)	0.138
Organic food store	3.23 (0.98)	2.61 (0.80)	**10.77 (6.77)**	**11.81 (2.25)**	5.74 (1.12)	**<0.001**
Specialized food stores^c^	7.45 (1.58)	8.42 (1.41)	**10.39 (4.08)**	**14.25 (2.34)**	**14.63 (2.23)**	**0.001**
Market	3.15 (0.71)	2.33 (0.58)	6.74 (2.60)	7.97 (1.57)	**11.38 (2.08)**	**<0.001**
Frozen food store	1.32 (0.33)	2.11 (0.77)	0.66 (0.38)	2.77 (0.70)	2.43 (0.53)	**0.023**
Online purchasing	0.57 (0.21)	1.59 (0.61)	0.77 (0.47)	0.88 (0.42)	0.91 (0.46)	0.430
Other	0.09 (0.04)	0.20 (0.11)	0.01 (0.01)	0.78 (0.42)	0.20 (0.07)	**<0.001**

*Share of expenses were calculated using till receipts collected during 1 month in the Mont'Panier study.*

a *E-supermarket: Online food shopping with pickup at supermarket (called drive in French).*

b *Producer: direct sales from producers [e.g., fruit and vegetable growers (called maraîchers in French), farmers, basket orders from Associations for the Maintenance of Peasant Agriculture (AMAP), which is a French version of Community Supported Agriculture].*

c *Specialized food stores: greengrocers, bakeries, butcher's, fishmonger's, and dairy stores.*

*The numbers in bold represent the means among each cluster that were >10% or p-values <0.05 for the last column*.

*Cluster* “*Supermarket*” was composed of households who did most of their grocery shopping in supermarkets, but reduced trips to, and increased quantity of food purchased in supermarkets during lockdown. Half of households of this cluster were composed of single adults, more than one-third of households were in the lowest income group (<980 €/month), and nearly half of household heads were over 50 years old and had an undergraduate degree. Mean share of expenses before lockdown was highest for supermarkets and discount grocery stores.

*Cluster* “*E-supermarket*” was composed of households who increased in frequency and in quantity purchased their use of online food shopping with pickup at supermarket. Very few households of this cluster reported never having used e-supermarkets before the lockdown. This cluster is mostly composed of households with multiple adults and at least one child, household of middle-low income (980–1,722 €/month) and household heads who were 35–50 years old and had an undergraduate degree. Mean share of expenses before lockdown was here again highest for supermarkets and discount grocery stores.

*Cluster* “*Producer*” included households who regularly purchased food directly from producers, but mostly reduced these purchases during lockdown. One-third of households of this cluster was composed of one adult with at least one child, had lower income (<980 €/month), nearly half of household heads were above 50 years old, and most had at least an undergraduate degree. Mean share of expenses before lockdown were about 10% for organic food stores, producers, specialized food stores, and small grocery stores; supermarkets had once again the most important share of expenses.

*Cluster* “*Organic food store*” was mostly composed of organic food store users, who did not change their frequency of use and quantity of food purchased in organic food stores during the lockdown. Most represented households were households composed of one adult, with higher income (>2,551 €/month), older and highly educated household heads (>50 years old and postgraduate degree). Besides supermarkets, most important shares of expenses before lockdown were for specialized food stores and organic food stores.

*Cluster* “*Diversified*” included households who had diversified food supply sources with reduced frequency of trips to supermarkets, markets, organic food stores, greengrocers and other specialized food stores and reduced quantity of food purchased in markets. This cluster was mostly composed of single adults, with higher income (>2,550 €/month), older and highly educated household heads (>50 years old and postgraduate degree). Most important shares of expenses before lockdown were for supermarkets, specialized food stores, and markets.

### Associations Between Cluster Membership and Individual and Environmental Factors

Results of multivariate logistic regression models assessing the associations between clusters of change in FPPs during lockdown and socioeconomic and demographic characteristics, perceived, and objective FE indicators are represented in Table 3.

**Table 3:** Associations between clusters and socioeconomic and demographic characteristics, perceived, and objective FE indicators.

	**Cluster Supermarket 38%**		**Cluster E-supermarket^a^ 12%**		**Cluster producer^b^ 8%**		**Cluster organic food store 20%**		**Cluster diversified 22%**	
	**OR**	**95% CI**	**OR**	**95% CI**	**OR**	**95% CI**	**OR**	**95% CI**	**OR**	**95% CI**
**Socioeconomic and demographic characteristics**										
**Household composition**										
One adult	—	—	—	—	—	—	—	—	—	—
Multiple adults	1.00	0.49, 2.07	0.62	0.20, 1.86	**4.30**	**1.02, 18.1**	1.08	0.48, 2.46	0.83	0.36, 1.90
One adult with at least one child	0.53	0.12, 2.34			**24.8**	**3.53, 175**	1.05	0.23, 4.80	1.05	0.27, 4.09
Multiple adults with at least one child	0.43	0.18, 1.03	1.36	0.54, 3.40	**7.47**	**1.25, 44.6**	0.55	0.22, 1.38	1.11	0.47, 2.61
**Income per consumption unit**										
<980 €/month	—	—	—	—	—	—	—	—	—	—
980–1,722 €/month	0.55	0.22, 1.37	**10.5**	**2.17, 51.2**	0.71	0.17, 2.92	0.94	0.27, 3.30	0.92	0.30, 2.83
1,723–2,550 €/month	0.37	0.12, 1.12	**8.06**	**1.69, 38.6**	0.54	0.12, 2.41	1.70	0.48, 5.97	1.48	0.44, 5.01
≥2,551 €/month	**0.28**	**0.08, 0.90**	**7.95**	**1.70, 37.3**	0.63	0.13, 2.92	1.67	0.47, 5.87	1.37	0.38, 5.03
**Age of household head**										
<35 years	—	—	—	—	—	—	—	—	—	—
35–50 years	0.61	0.22, 1.69	0.77	0.26, 2.32	0.49	0.07, 3.53	**3.53**	**1.08, 11.5**	0.69	0.24, 1.96
>50 years	0.81	0.34, 1.94	0.45	0.15, 1.34	2.95	0.68, 12.9	2.35	0.81, 6.85	0.55	0.21, 1.45
**Level of education of household head**										
High school degree or lower	—	—	—	—	—	—	—	—	—	—
Undergraduate degree	0.69	0.27, 1.73	0.68	0.20, 2.35	7.60	0.91, 63.1	1.58	0.52, 4.76	0.63	0.22, 1.78
Postgraduate degree	0.62	0.22, 1.72	0.38	0.10, 1.37	4.53	0.53, 38.3	1.77	0.59, 5.34	1.39	0.49, 3.93
**Median income (IRIS or municipality)**										
Low			—	—					—	—
Medium-low			0.55	0.18, 1.67					2.07	0.69, 6.17
Medium-high			1.75	0.62, 4.97					0.57	0.16, 1.98
High			0.59	0.21, 1.66					2.30	0.89, 5.96
**Perceived food environment**										
**Distance from home to the closest general food store**										
<5 min	—	—	—	—						
Between 5 and 15 min	1.63	0.73, 3.61	1.73	0.77, 3.88						
Between 15 and 30 min	2.68	1.00, 7.18	**0.17**	**0.05, 0.61**						
More than 30 min	**4.74**	**1.32, 17.0**								
**Perception of increased food prices during the lockdown**										
No	—	—			—	—	—	—	—	—
Yes	**2.44**	**1.10, 5.39**			**0.04**	**0.01, 0.35**	**0.16**	**0.07, 0.40**	**3.23**	**1.23, 8.49**
Don't know	1.95	0.89, 4.29			1.17	0.39, 3.46	**0.27**	**0.13, 0.58**	2.14	0.87, 5.28
**In-store availability of food products (reason for change** ^**c**^ **)**										
No			—	—	—	—	—	—		
Yes			**2.91**	**1.33, 6.36**	2.27	0.81, 6.40	**0.42**	**0.18, 0.98**		
**Buying local products (reason for change^c^)**										
No	—	—			—	—			—	—
Yes	**0.30**	**0.15, 0.62**			**0.17**	**0.03, 0.90**			**3.46**	**1.71, 7.02**
**Objective food environment**										
**Presence of an organic food store (1,000 m from home)**										
No	—	—			—	—				
Yes	**0.25**	**0.09, 0.72**			**5.46**	**1.86, 16.1**				

*Only FE indicators associated with clusters at 0.1 significance level in bivariate analyses were retained for inclusion in multivariate models.*

*Multivariable backwards-stepwise logistic regression was performed to determine the variables included in the final model, with income per unit of consumption, household structure, age, and educational level of household head forced into the model.*

a *E-supermarket: Online food shopping with pickup at supermarket (called drive in French).*

b *Producer: direct sales from producers [e.g., fruit and vegetable growers (called maraîchers in French), farmers, basket orders from Associations for the Maintenance of Peasant Agriculture (AMAP), which is a French version of Community Supported Agriculture].*

c *As a reason for change in FPPs during the lockdown.*

*The numbers in bold represent significant results (p < 0.05).*

Compared to other clusters, households belonging to the *Cluster* “*Supermarket*” were less likely to have higher incomes, but more likely to live at more than 30 min from a general food store and to perceive a rise in food prices during lockdown. They were also less likely to report “buying local products” as a reason for change in FPPs and to live within a 1-km walking distance from an organic food store.

Households from *Cluster* “*E-Supermarket*” were more likely to have higher incomes, less likely to live at 15 min or more from a general food store and more likely to report “in-store availability of food products” as a reason for change in FPPs.

In *Cluster* “*Producer*” were households who were less likely to be composed of a single adult, to perceive a rise in food prices during lockdown and to report “buying local products” as a reason for change in FPPs; households were however more likely to live within a 1-km walking distance from an organic food store.

Households belonging to *Cluster* “*Organic food store*” were more likely to have an older household head (35–50 vs. <35 years old), less likely to perceive a rise in food prices during lockdown and to report “in-store availability of food products” as a reason for change in FPPs.

Households from *Cluster* “*Diversified*” were more likely to perceive a rise in food prices during lockdown and to report “buying local products” as a reason for change in FPPs.

Drop of income during lockdown and store accessibility (closure, public transportation, and parking facilities, etc.) as a reason for change in FPPs during lockdown had *p*-values > 0.1 in bivariate analysis and were thus not included in multivariate models. Not cited socioeconomic and demographic characteristics, perceived FE variables and also most objective FE indicators were not statistically significantly associated with given clusters.

## Discussion

By exploring changes in FPPs of French households during the first COVID-19 lockdown and their related individual and environmental factors, our study highlighted diverse grocery shopping practices with a global tendency of reduced frequency of trips to food outlets, but no major change in food outlet choice. Significant associations of these practices with sociodemographic characteristics and perceived FE indicators were also found, rather than with objective FE indicators.

Despite the expected rise in popularity of alternative food supply chains, which were widely covered in the press during the COVID-19 crisis and spontaneously evoked by involved French consumers during the lockdown (19), our study rather suggests a modest increase in new users of alternative food supply sources such as producers and a persistent dominance of the industrial food system, leading with supermarkets, as the main food supply source for consumers.

More precisely, results of our study show that frequency of trips to food stores tends to have globally reduced during the lockdown, with the exception of e-supermarkets (online food shopping with pickup at supermarket), which were more frequently used by households during this period; similar results were found in another French study (8). Likewise, it has been reported elsewhere that during the lockdown, consumers reduced shopping trips and concentrated most food purchases on one shop, and thus, supermarket sales went up at the costs of other retail outlets (17). Frequency of trips has probably been reduced to limit exposure to the COVID-19 virus, which was the most frequently reported reason for changes in food supply sources in our study.

Before the lockdown, households did most of their grocery shopping in supermarkets, and mean share of expenses was highest for supermarkets in all five clusters, but especially for *Clusters* “*Supermarket*” and “*E-supermarket*,” in which households spent more than half of their expenses in supermarkets. These two clusters, which include 50% of households of our total sample, differentiated their FPPs during the lockdown by either sticking to supermarkets with reduced frequency of trips and increased quantity purchased, or by increasing their use of online food purchasing with pickup at supermarket. Lower income households seem to have chosen to stick to traditional stationary shopping, whereas higher income households seem to have turned to online food shopping. These results seem coherent, since lower income households are less inclined to make use of e-grocery shopping practices (27), which is not surprising given that ownership of computing equipment with internet access such as smartphones, tablets, laptops, and computers, needed for online purchasing, goes together with higher incomes (28). This brings out some social inequalities regarding food purchasing opportunities for lower income households.

Results of multivariate analysis showed that households of *Cluster* “*Supermarket*,” compared to households of other clusters, were more likely to live at more than 30 min from a general food store and to perceive a rise in food prices during lockdown. Living further away from a general food store nudges the consumer to concentrate most food shopping in one place and the supermarket, which offers a variety of food products, allows the consumer to find all he needs at once. Perception of increased food prices is most likely more important for lower income households, since they pay more attention to price fluctuations and a food price inflation due to COVID-19 restrictions has indeed been observed in Europe during the first lockdown period (March to April 2020) (21). *Cluster* “*Supermarket*” was negatively associated with “buying local products” as a reason for change in FPPs. These results are coherent with those of a study conducted in France, which suggest that consumers shopping mainly in supermarkets are less likely to be involved with local food production (29).

Belonging to the *Cluster*, “*E-supermarket*” was associated with reporting “in-store availability of food products” as a reason for changes in FPPs. In-store availability of food produces has been identified as an issue in a great number of supermarkets during COVID-19 lockdown, mainly due to consumer's stockpiling behavior (22, 23). It is thus not surprising that households who before lockdown used to do most of their grocery shopping in supermarkets turned to online food shopping partly because of lower in-store availability of food products. The advantage of e-supermarkets being that you are aware beforehand of produce availability, which allows you to avoid wasting a trip to the supermarket for nothing.

As opposed to the two above cited clusters, *clusters* “*Diversified*,” “*Organic Food Store*,” and “*Producer*” had more diversified food-shopping sources. Even though share of expenses before lockdown was also most important for supermarkets, it was of about 10–15% for other food stores, which includes specialized food stores and markets for *cluster* “*Diversified*,” specialized food stores and organic food stores for *cluster* “*Organic food store*” and specialized food stores, organic food stores, greengrocers, and small grocery store for *cluster* “*Producer*.”

*Cluster* “*Diversified*” included households who had diversified food supply sources, but who reduced frequency of trips to supermarkets, markets, organic food stores, greengrocers, and other specialized food stores during the lockdown and also reduced quantity of food purchased in markets. Households of this cluster were more likely to report “buying local products” as a reason for change in FPPs. Varying food store types might be a way for the consumer to find local food products produced by small local firms (30). As a matter of fact, greengrocers and other specialized food stores, which include bakeries, butcher's, fishmongers, dairy stores, are settings in which the consumer is able to ask where the food comes from and how it had been produced, as opposed to supermarkets where the staff in contact with consumers has no role in the production or supply of products (29). Moreover, households of this cluster were more likely to perceive a rise in food prices during lockdown. Even though there was no significant association with income level, descriptive analysis showed that 43% of households of this cluster had lower income and were thus probably more prone to perceive price variations.

*Cluster* “*Organic food store*” is mostly composed of households who had few changes in their FPPs, which were diversified in food supply sources, but more substantial for organic food stores. This cluster included households who were more likely to have an older household head (35–50 vs. <35 years old), less likely to report “in-store availability of food products” as a reason for change in FPPs and less likely to perceive a rise in food prices during lockdown. Likewise, another study also found that those aged 35–44 had a higher probability of consuming organic products (31). Given that stockpiling during lockdown was mostly noted in supermarkets (since they were the most frequented food outlets), in-store availability of food products might not have been as noticeable for consumers who frequented other food store types. Moreover, consumers who prefer organic food products tend to be less price sensitive (32).

Households of *cluster* “*Producer*,” who reduced purchases from producers during the lockdown, were more likely to live within a 1-km walking distance from an organic food store. This seems coherent, since two out of three households of this cluster are organic food stores users (only 35.7% reported never using them). One could hypothesize that those households replaced their purchases from producers with purchases from organic food stores, probably because of accessibility issues, which was reported as a reason for change in FPPs for 46.3% of households of this cluster. Additionally, indeed, open air street stands, which are often used by producers to sell their products on the side of the road, were closed during the first lockdown in France. Households of *cluster* “*Producer*” were also less likely to be composed of a single adult, to perceive a rise in food prices during lockdown and to report “buying local products” as a reason for change in FPPs. As stated by another study conducted in France, consumers of less traditional food retailers (as opposed to traditional supermarket users) are less price-sensitive, probably because they are aware of the cost of the production process and may consider price as an indicator of quality (29). In addition, these households were already invested in buying local products before the lockdown; reasons for change in FPPs which involved reducing purchases from producers were thus not to buy local products. It should be noted that being constituted of 8% of households of the total sample, this cluster is the smallest of the five identified clusters of our study sample, and results are thus to be considered with caution.

Drop of income during lockdown and store accessibility (closure, public transportation, and parking facilities, etc.) as a reason for change in FPPs and most objective FE indicators were not statistically significantly associated with any of the five identified clusters of our study. Percentage of households who reported a drop of income during lockdown was relatively well-distributed among the five clusters, the same counts for store accessibility as a reason for change in FPPs. Objective FE, also called community or built FE, often presents less consistent significant relationships with dietary behaviors than perceived FE (33). Indeed, objective FE indicators on their own simply cannot capture non-geographic dimensions of the FE (33), such as in-store availability of food products, food prices, and consumer's preferences.

### Strengths and Limitations

The strengths of our study include the timing of the data collection to capture changes during the lockdown, which was launched in April of 2020, so *during* the first COVID-19 related lockdown and not after, thereby limiting memory bias of participants. In addition, comparisons were possible with objectively measured food purchasing behaviors before the lockdown (e.g., share of expenses by food outlet type) due to the original data set of the Mont'Panier study which collected details of households' food supply before the lockdown (May 2018 to December 2019) using food purchase receipts. Another strength is the use of both perceived and objective FE indicators. The importance of combining both perceived and objective FE measures has previously been highlighted in a systematic review (33), where authors point to the fact that studies should not only take into account the geographical aspects of the FE, but also in-store availability of food products, food prices, and consumer's preferences (33).

We acknowledge that there were some limitations to this study. First, caution is needed regarding the extrapolation of these results to the entire French population, since this study was limited to a metropolitan area located in the South of France. Results would most likely be different in a less densely populated urban area or in a rural setting. Moreover, changes in food shopping behaviors and related variables were self-reported, and thus, misreporting may have occurred, however to account (at least at some extent) for this potential bias, comparisons between data collected before and during the lockdown were carried out. For instance, frequency of use of food supply sources before lockdown, obtained through the 1-month collection of receipts in the Mont'Panier study, was compared to the reported changes in frequency of use of food supply sources during the lockdown. Sample size is another limitation of our study, thereby limiting the validity and generalizability of our study's results. Finally, selection bias may also be an issue in this study, since households of this study were mostly highly educated. However, to limit this selection bias, quota sampling was performed based on household composition crossed with age of household head, plus all analyses presented in this paper were conducted on a weighted sample, which was adjusted by calibration on margins based on income per unit of consumption and household composition crossed with household head's age group.

## Conclusion

In conclusion, this study highlighted diverse changes in FPPs of southern French households during the first COVID-19 lockdown and some associations between these changes and related individual and environmental factors. Overall, our results showed more significant associations with perceived than with objective FE indicators, which highlights the importance of combining both measures when assessing relationships with dietary behaviors. Better understanding FPPs and associated FE characteristics are important, especially now given the exacerbated food retail access concerns that came along with the COVID-19 pandemic.

Despite the expected rise in popularity of short supply chains, the obvious ongoing supremacy of supermarkets in the food retail sector and the shift from stationary to online food shopping highlighted in our study show that there is still room for improvement to create a more sustainable and resilient food system. For future lockdowns, public health policies and city councils should consider strengthening online food purchasing, since they help avoid physical contact and reduce thus the risk of new infections. For more sustainable urban food systems, innovations in safe grocery shopping practices for short supply chains, small food outlets, and local producers should be encouraged by policy makers. Given our findings on social inequalities regarding food purchasing opportunities for low-income populations, special efforts should be made to find new ways to increase safe access to food for those with no internet access and no car. Possible strategies to consider include expanding or implementing food purchases through phone orders, possibility for pedestrian pickup, and free home delivery services.

Urban food planning policies should take into account the diversification of food purchasing opportunities that seem to have occurred during this first lockdown and pay close attention to a potential social fragmentation in FPPs. Beyond the pandemic, results of this study might thus provide useful information for cities looking to improve their FE in the long run.

## Data Availability Statement

Restrictions apply due to the protection of health data regulation set by the French National Commission on Informatics and Liberty (Commission Nationale de l'Informatique et des Libertés, CNIL). Requests to access these datasets should be directed to Caroline Méjean, caroline.mejean@inrae.fr.

## Ethics Statement

The studies involving human participants were reviewed and approved by the Institutional Review Board of the French Institute for Health and Medical Research (IRB Inserm n° IRB00003888 IORG0003254 FWA00005831) and were registered to the Commission Nationale Informatique et Libertés. This study was conducted according to the guidelines laid down in the Declaration of Helsinki. The patients/participants provided their written informed consent to participate in this study.

## Surfood-Foodscapes Working Group

The following authors were part of the Surfood-Foodscapes Working Group: Caroline Mejean, Christophe Soulard, Coline Perrin, Daisy Recchia, Emmanuelle Cheyns, Géraldine Chaboud, Marion Tharrey, Marlène Perignon, Nicolas Bricas, Nicole Darmon, Olivier Lepiller, Pascale Sheromm, Pascaline Rollet, and Simon Vonthron.

## Author Contributions

CM, MP, and NB designed the study and developed the questionnaire and the protocol for data collection. PR performed data management and undertook data analysis. SV and CP calculated FE indicators. DR wrote the first draft of the manuscript. Surfood-Foodscapes Working Group gave insights on the interpretation of the results. All authors contributed to manuscript revision, read, and approved the submitted version.

## Funding

This work was carried out as part of DR's Ph.D. funded by Région Occitanie and Institut National de Recherche pour l'Agriculture, l'Alimentation et l'Environnement (INRAE). The project Sustainable Urban Food Systems—the effects of urban foodscape on food styles in Montpellier Metropole (Surfood-Foodscapes) coordinated by Cirad, Inrae, and Montpellier Supagro, was publicly funded through ANR (the French National Research Agency) under the Investissements d'Avenir programme with the reference ANR-10-LABX-001-01 Labex Agro and coordinated by Agropolis Fondation. The project Mont'Panier Relations entre paysages alimentaires et pratiques alimentaires, was also funded by Région Occitanie, Dispositif REVE REcherche et Valorisation Economique. The funders had no role in the study design, data collection and analysis, decision to publish, or preparation of this manuscript.

## Conflict of Interest

The authors declare that the research was conducted in the absence of any commercial or financial relationships that could be construed as a potential conflict of interest.

## Publisher's Note

All claims expressed in this article are solely those of the authors and do not necessarily represent those of their affiliated organizations, or those of the publisher, the editors and the reviewers. Any product that may be evaluated in this article, or claim that may be made by its manufacturer, is not guaranteed or endorsed by the publisher.
