# Corrigendum: Changes in Food Purchasing Practices of French Households During the First COVID-19 Lockdown and Associated Individual and Environmental Factors

**DOI:** 10.3389/fnut.2022.925426

**Published:** 2022-05-24

**Authors:** Daisy Recchia, Pascaline Rollet, Marlène Perignon, Nicolas Bricas, Simon Vonthron, Coline Perrin, Caroline Méjean

**Affiliations:** ^1^MoISA, Univ Montpellier, CIRAD, CIHEAM-IAMM, INRAE, Institut Agro, IRD, Montpellier, France; ^2^CIRAD, UMR MoISA, Montpellier, France; ^3^INNOVATION, Univ Montpellier, CIRAD, INRAE, Institut Agro, Montpellier, France

**Keywords:** COVID-19 lockdown, food purchasing behaviors, grocery shopping, food outlets, food environment, France

In the published article, there was an error regarding the affiliation(s) for “Simon Vonthron and Coline Perrin.” Instead of affiliation 2, they should have affiliation 3 “INNOVATION, Univ Montpellier, CIRAD, INRAE, Institut Agro, Montpellier, France.”

The authors apologize for this error and state that this does not change the scientific conclusions of the article in any way. The original article has been updated.

## Publisher's Note

All claims expressed in this article are solely those of the authors and do not necessarily represent those of their affiliated organizations, or those of the publisher, the editors and the reviewers. Any product that may be evaluated in this article, or claim that may be made by its manufacturer, is not guaranteed or endorsed by the publisher.

