# Supplementary material for: Changes in Food Purchasing Practices of French Households During the First COVID-19 Lockdown and Associated Individual and Environmental Factors
Source: Front Nutr. 2022 Mar 3;9:828550. doi: 10.3389/fnut.2022.828550 (PMC8928476; doi:10.3389/fnut.2022.828550)
Supplement: Supplementary file 1 [file Table_1.DOCX]

Supplementary Material

**Supplementary Table 1.** Main reasons for change in food purchasing practices during lockdown, perceived food environment and related factors

|  | **Total sample^1^** | **Cluster Supermarket 38%** | **Cluster E-supermarket^2^ 12%** | **Cluster Producer^3^ 8%** | **Cluster Organic food store 20%** | **Cluster Diversified  22%** | **Pearson's X² p-value** |
| --- | --- | --- | --- | --- | --- | --- | --- |
| **Main reasons for change in FSP during lockdown** | |  |  |  |  |  |  |
| Limit exposure to COVID-19 | **66.4%** | **65.6%** | **70.3%** | **62.8%** | **54.4%** | **77.8%** | 0.256 |
| Cooking and consumption change | **34.1%** | 28.3% | 29.2% | **34.8%** | **44.9%** | **36.8%** | 0.443 |
| Distance to food store | 31.1% | 29.2% | 25.3% | 25.9% | 24.1% | **46.0%** | 0.222 |
| Availability of products in store | 29.7% | 28.8% | **51.4%** | **48.2%** | 14.6% | 26.4% | **0.017** |
| Store accessibility^4^ | 29.4% | 23.5% | **37.3%** | **46.3%** | 30.8% | 27.9% | 0.384 |
| Buy local products | 27.8% | 13.9% | 30.9% | 7.7% | **35.4%** | **50.5%** | **<0.001** |
| Opportunity to go outdoors | 20.2% | 20.0% | 26.8% | 11.4% | 18.4% | 21.5% | 0.8094 |
| Reorganization of daily routines | 16.0% | 11.6% | 14.8% | 7.4% | 25.1% | 18.9% | 0.2428 |
| Budget constraints | 8.3% | 12.8% | 7.9% | 4.2% | 0.7% | 8.8% | 0.119 |
| No change | 9.2% | 3.7% | 8.1% | 4.0% | 29.3% | 3.0% | **<0.001** |
| **Distance from home to the closest general food store** |  |  |  |  |  |  | **0.014** |
| Less than 5 minutes | 31.5% | 18.7% | 35.0% | **58.9%** | 33.5% | **40.4%** |  |
| Between 5 and 15 minutes | **38.1%** | **32.1%** | **57.3%** | 35.8% | **40.9%** | 35.8% |  |
| Between 15 and 30 minutes | 17.8% | 24.5% | 6.6% | 5.3% | 17.0% | 17.6% |  |
| More than 30 minutes | 10.6% | 19.7% | 0.0% | 0.0% | 8.6% | 6.2% |  |
| Don’t know | 2.1% | 5.0% | 1.2% | 0.0% | 0.0% | 0.0% |  |
| **Perception of increased food prices during lockdown** | |  |  |  |  |  | **<0.001** |
| Yes | 33.0% | 42.4% | 32.2% | 3.8% | 16.5% | 42.1% |  |
| No | 21.2% | 12.6% | 16.3% | 24.8% | **48.6%** | 12.9% |  |
| Don’t know | **45.8%** | **45.0%** | **51.6%** | **71.4%** | 35.0% | **45.0%** |  |
| **Stockpiling during lockdown** |  |  |  |  |  |  | 0.538 |
| Yes | 31.1% | 29.7% | 43.9% | 20.8% | 23.7% | 36.5% |  |
| No | **68.5%** | **70.3%** | **56.1%** | **79.2%** | **75.4%** | **62.3%** |  |
| Don’t know | 0.5% | 0.0% | 0.0% | 0.0% | 0.9% | 1.2% |  |
| **Change of total amount of grocery expenses during lockdown** | |  |  |  |  |  | 0.161 |
| Increased | **54.2%** | **54.4%** | **74.7%** | 43.1% | **55.6%** | 44.6% |  |
| Did not change | 30.6% | 28.0% | 16.4% | **47.8%** | 34.1% | 33.9% |  |
| Decreased | 9.1% | 7.6% | 8.9% | 0.0% | 6.1% | 18.0% |  |
| Don’t know | 6.1% | 10.0% | 0.0% | 9.1% | 4.2% | 3.5% |  |
| **Drop of households’ income during lockdown** |  |  |  |  |  |  | 0.703 |
| Yes | 27.0% | 29.3% | 34.1% | 16.4% | 24.3% | 25.1% |  |
| No | **72.0%** | **70.2%** | **64.6%** | **81.7%** | **73.3%** | **74.9%** |  |
| Don’t know | 1.0% | 0.5% | 1.2% | 1.9% | 2.5% | 0.0% |  |

^1^The sample was adjusted by calibration on margins based on income per unit of consumption and household composition crossed with household head’s age group.

^2^E-supermarket: Online food shopping with pick up at supermarket (called *drive* in French).

^3^Producer: direct sales from producers (e.g. fruit and vegetable growers (called *maraîchers* in French), farmers, basket orders from Associations for the Maintenance of Peasant Agriculture (AMAP), which is a French version of Community Supported Agriculture).

^4^Store accessibility: Closure, public transportation and parking facilities, etc.

The numbers in bold represent the highest percentages among each cluster for each variable, except for main reasons for change in FSP during lockdown, where they represent percentages >33% and last column where they represent p<0.05.

**Supplementary table 2.** Changes in food purchasing practices during lockdown, namely frequency of use and quantity purchased per food supply source

|  | **Cluster  Supermarket  38%** | | **Cluster  E-supermarket^1^  12%** | | **Cluster  Producer^2^  8%** | | **Cluster  Organic Food Store**  **20%** | | **Cluster  Diversified  22%** | |
| --- | --- | --- | --- | --- | --- | --- | --- | --- | --- | --- |
|  | **Frequency** | **Quantity** | **Frequency** | **Quantity** | **Frequency** | **Quantity** | **Frequency** | **Quantity** | **Frequency** | **Quantity** |
| **Supermarket** |  |  |  |  |  |  |  |  |  |  |
| More | 21.8% | **53.1%** | 14.6% | 10.6% | 4.2% | 18.2% | 5.2% | 19.4% | 8.2% | 34.3% |
| Same | 13.0% | 25.0% | 2.7% | 9.6% | 23.2% | 45.7% | 33.7% | 35.0% | 15.6% | 20.8% |
| Less | **57.7%** | 15.6% | 36.6% | 43.1% | 45.4% | 9.0% | 25.4% | 18.2% | **59.1%** | 29.3% |
| Never / not applicable | ***7.5%*** | ***6.3%*** | 46.1% | 36.8% | 27.2% | 27.2% | 35.7% | 27.4% | 17.1% | 15.6% |
| **E-Supermarket^1^** |  |  |  |  |  |  |  |  |  |  |
| More | 0.7% | 0.4% | **62.3%** | **50.9%** | 4.4% | 4.4% | 4.4% | 5.2% | 3.0% | 3.1% |
| Same | 0.0% | 1.5% | 31.1% | 43.3% | 1.6% | 1.6% | 1.6% | 3.0% | 0.0% | 0.8% |
| Less | 5.6% | 5.9% | 3.9% | 5.8% | 6.9% | 6.9% | 9.9% | 5.7% | 12.3% | 13.0% |
| Never / not applicable | 93.7% | 92.2% | ***2.7%*** | ***0.0%*** | 87.0% | 87.0% | 84.1% | 86.1% | 84.7% | 83.2% |
| **Producer^2^** |  |  |  |  |  |  |  |  |  |  |
| More | 7.4% | 6.8% | 24.8% | 20.6% | 0.0% | 0.0% | 33.9% | 21.6% | 26.4% | 23.9% |
| Same | 2.6% | 0.7% | 2.4% | 3.6% | 0.0% | 2.1% | 15.6% | 25.6% | 6.7% | 5.8% |
| Less | 0.0% | 0.0% | 0.0% | 7.9% | **98.0%** | **93.3%** | 1.4% | 1.0% | 1.6% | 4.9% |
| Never / not applicable | 90.0% | 92.5% | 72.8% | 67.9% | ***2.0%*** | ***4.6%*** | 49.1% | 51.8% | 65.3% | 65.4% |
| **Organic food store** |  |  |  |  |  |  |  |  |  |  |
| More | 3.8% | 5.2% | 0.9% | 4.3% | 21.5% | 27.7% | 12.0% | 19.5% | 11.7% | 45.7% |
| Same | 3.4% | 5.0% | 18.0% | 5.4% | 8.5% | 1.9% | **68.1%** | **71.3%** | 12.6% | 9.3% |
| Less | 16.7% | 13.7% | 31.7% | 46.6% | 34.3% | 34.7% | 16.9% | 7.2% | **63.1%** | 37.1% |
| Never / not applicable | 76.1% | 76.1% | 49.4% | 43.8% | 35.7% | 35.7% | ***3.0%*** | ***2.1%*** | 12.5% | ***7.9%*** |
| **Market** |  |  |  |  |  |  |  |  |  |  |
| More | 7.3% | 2.5% | 0.0% | 6.2% | 9.4% | 7.1% | 8.1% | 13.6% | 5.1% | 0.9% |
| Same | 0.7% | 0.6% | 4.8% | 2.4% | 0.0% | 7.8% | 31.6% | 28.4% | 0.0% | 1.2% |
| Less | 9.1% | 13.5% | 20.9% | 23.2% | 33.0% | 26.0% | 33.4% | 30.9% | **64.9%** | **70.0%** |
| Never / not applicable | 82.9% | 83.4% | 74.4% | 68.3% | 57.7% | 59.1% | 26.9% | 27.2% | 30.0% | 28.0% |
| **Greengrocer** |  |  |  |  |  |  |  |  |  |  |
| More | 10.8% | 20.4% | 8.4% | 13.8% | 13.3% | 21.0% | 17.6% | 26.1% | 28.2% | 43.1% |
| Same | 10.1% | 8.8% | 19.0% | 15.2% | 1.8% | 9.4% | 41.1% | 33.2% | 5.7% | 21.9% |
| Less | 24.0% | 13.3% | 22.7% | 27.0% | 43.5% | 28.2% | 3.4% | 2.6% | **57.4%** | 29.0% |
| Never / not applicable | 55.1% | 57.6% | 49.9% | 44.1% | 41.4% | 41.4% | 37.8% | 38.2% | ***8.7%*** | ***6.0%*** |
| **Specialized food stores^3^** |  |  |  |  |  |  |  |  |  |  |
| More | 4.2% | 13.8% | 20.9% | 16.8% | 12.0% | 20.6% | 16.2% | 16.9% | 14.1% | 22.5% |
| Same | 13.3% | 13.4% | 9.0% | 17.2% | 20.0% | 21.2% | 33.5% | 42.2% | 12.4% | 15.1% |
| Less | 13.0% | 2.9% | 17.3% | 14.7% | 23.8% | 13.0% | 24.4% | 17.2% | **56.7%** | 49.9% |
| Never / not applicable | 69.6% | 69.9% | 52.8% | 51.3% | 44.3% | 45.2% | 25.9% | 23.8% | 16.8% | 12.5% |
| **Small grocery store** |  |  |  |  |  |  |  |  |  |  |
| More | 8.6% | 4.4% | 13.2% | 8.5% | 5.6% | 0.0% | 8.3% | 6.5% | 29.2% | 36.7% |
| Same | 11.7% | 12.7% | 2.5% | 4.0% | 2.1% | 15.5% | 19.9% | 22.6% | 4.8% | 13.3% |
| Less | 7.1% | 12.2% | 6.0% | 14.2% | 42.7% | 36.8% | 13.4% | 15.3% | 39.6% | 27.5% |
| Never / not applicable | 72.6% | 70.8% | 78.3% | 73.3% | 49.7% | 47.7% | 58.4% | 55.7% | 26.4% | 22.5% |
| **Discount food store** |  |  |  |  |  |  |  |  |  |  |
| More | 6.6% | 8.0% | 1.2% | 6.6% | 0.0% | 0.0% | 1.0% | 1.0% | 0.9% | 0.9% |
| Same | 13.4% | 12.2% | 9.4% | 3.9% | 2.1% | 2.2% | 14.8% | 18.7% | 12.2% | 13.1% |
| Less | 25.2% | 20.2% | 31.2% | 35.5% | 10.4% | 3.7% | 9.6% | 6.0% | 28.9% | 25.6% |
| Never / not applicable | 54.8% | 59.6% | 58.2% | 54.0% | 87.6% | 94.1% | 74.6% | 74.3% | 58.0% | 60.4% |
| **Frozen food store** |  |  |  |  |  |  |  |  |  |  |
| More | 1.9% | 3.4% | 11.9% | 10.3% | 6.9% | 0.0% | 7.5% | 9.3% | 2.7% | 5.6% |
| Same | 2.9% | 3.9% | 12.8% | 15.6% | 0.0% | 2.1% | 35.1% | 31.5% | 12.2% | 16.3% |
| Less | 5.7% | 2.7% | 7.4% | 12.5% | 1.6% | 10.6% | 0.9% | 5.4% | 41.3% | 38.1% |
| Never / not applicable | 89.5% | 90.0% | 67.8% | 61.6% | 91.4% | 87.4% | 56.5% | 53.7% | 43.8% | 40.0% |

^1^Cluster E-supermarket: online food shopping with pick up at supermarket (called *drive* in French).

^2^Producer: direct sales from producers (e.g. fruit and vegetable growers (called *maraîchers* in French), farmers, basket orders from Associations for the Maintenance of Peasant Agriculture (AMAP), which is a French version of Community Supported Agriculture).

^3^Specialized food stores: include butcher's, fishmonger's and dairy stores.

The numbers in bold represent the highest percentages among each cluster (for modalities more, same and less) and the numbers in bold and italics represent the lowest percentages among each cluster (for modality never / not applicable); for cluster diversified multiple percentages were highlighted because multiple percentages were >50% or <10%.
